# Rapid Destructive Arthropathy of the Knee in Parkinson's Disease with Pisa Syndrome: A Case of Knee-Spine Syndrome

**DOI:** 10.1155/2021/6622445

**Published:** 2021-09-03

**Authors:** Hirokazu Takai, Masato Kitajima, Seiko Takai, Tomoki Takahashi, Ken-ichi Katsura, Makoto Tokunaga, Susumu Watanabe

**Affiliations:** ^1^Department of Orthopaedic Surgery, Kumamoto Kinoh Hospital, Japan; ^2^Department of Rehabilitation Medicine & Neurology, Kumamoto Kinoh Hospital, Kumamoto, Kumamoto, Japan

## Abstract

The changes occurring in knee osteoarthritis often cause alterations in the spinal loading condition, which further lead to degenerative changes. This close relationship of the knee and spine has been reported as knee-spine syndrome. A 60-year-old woman with Parkinson's disease (PD; Hoehn-Yahr stage IV) had severe knee pain with moderate lateral osteoarthritis of the knee (Kellgren-Lawrence classification grade II). Conservative therapy had no effect at all, and the knee developed destructive osteoarthritis rapidly without any traumatic episodes. The radiographic findings progressed to Kellgren-Lawrence grade IV within a month. Magnetic resonance imaging revealed partial depression of the joint surface, including shredded ossicles and substantial amounts of synovial fluid. The imaging findings were considered to be caused by a subchondral insufficiency fracture (SIF). Total knee arthroplasty was performed using a semiconstrained prosthesis. The alignment of her lower extremity improved, and the patient could walk without knee pain. The patient had Pisa syndrome, a lateral flexion of the trunk, which is a postural deformity of the trunk secondary to long-standing PD. The postural deformity in PD is not based on spinal deformity itself but on the loss of postural reflexes and the imbalance of muscle tonus. Her left knee pain appeared 1 month after L1-L4 posterior lumbar interbody fusion (PLIF) as the Pisa syndrome to her left side worsened. The more the trunk tilts to the lateral side, the center of the gravity axis will shift and pass through more lateral points of the knee and result in higher knee load. The stress concentration from the spine to the lateral joint of the knee caused lateral knee osteoarthritis, namely, knee-spine syndrome. When patients undergo correction surgery for adult spinal disorder with impairment of postural reflexes, they need to be followed up carefully regarding not only the spinal alignment but also the lower extremities.

## 1. Introduction

The spine and hip joints have significant effects on each other. Offierski and MacNab first identified this in 1983 and termed it hip-spine syndrome [1]. Several studies have reported the relation between spinal alignment disorder and hip osteoarthritis [2–4]. The changes caused by knee osteoarthritis also relate to the alterations in the spinal loading condition and degenerative changes. This close relationship between the knee and spine is termed knee-spine syndrome [5–8]. Strictly speaking, it is termed spine-knee syndrome because the postural deformity of the spine adversely affects the knee and worsens the osteoarthritis [8].

We present a case of subchondral insufficiency fracture (SIF) of the knee that was considered to be caused by spinal disorder. In this report, we describe a case of rapid destructive arthropathy of the knee in a patient with severe Parkinson's disease (PD) with Pisa syndrome.

## 2. Case History

### 2.1. Informed Consent

Written informed consent was obtained from the patient for publication of this case report and the accompanying images. A copy of the written consent form is available for review by the editor-in-chief of this journal on request.

### 2.2. Patient Information

**Figure 1:**
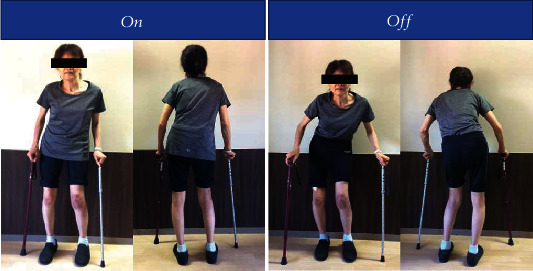
The picture of the PD patient standing on and off. The picture shows the appearance of the patient with PD. She managed to remain standing and walk leaning the body to her left side. The postural deformities—Pisa syndrome and bent posture—became noticeable when PD was off.

A 60-year-old woman (body weight: 45 kg, body mass index: 20.4 kg/m^2^) with severe PD presented with left knee pain. She was diagnosed with PD 12 years prior, and she had been medicated with several anti-PD medications. The symptoms of PD waxed and waned frequently. She often experienced the wearing-off phenomenon. Using a cane, she managed to remain standing and walk leaning the body to her left side. She had Pisa syndrome and scoliosis that were postural deformities of the trunk as symptoms of her PD (Figure 1). She required partial assistance throughout her daily life. Her Hoehn-Yahr scale was grade IV.

**Figure 2:**
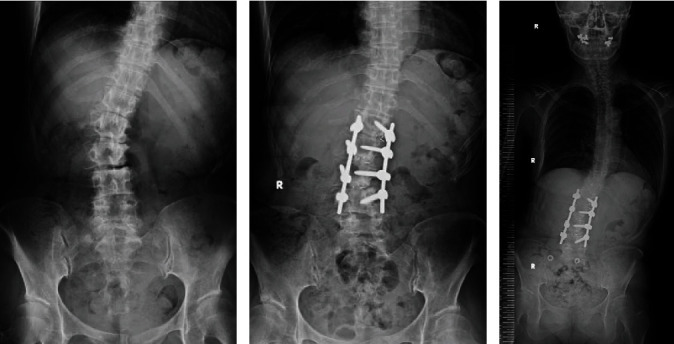
Radiographs of total spinal alignment. (a) Preoperative radiograph revealing lumbar spinal alignment in a supine position. (b) Postoperative radiograph of L1-L4 PLIF revealing lumbar spinal alignment in a supine position. (c) Radiograph showing total spinal alignment in a standing position. Pisa syndrome: the leaning of the body exacerbated when walking or standing but improved in the supine position.

Her medical history included L1-L4 PLIF for lumbar degenerative spondylolisthesis and scoliosis to get rid of her leg pain, which was performed in another hospital one month prior to the gradual onset of her left knee pain (Figures 2(a) and 2(b)). Her hip joints showed bilateral acetabular dysplasia without pain. Two months after the gradual onset of left knee pain, she visited an orthopedic clinic. Knee joint punctures and intra-articular hydrocortisone injections were performed several times. Betamethasone sodium phosphate was injected at the first visit, and triamcinolone acetonide was injected at the second one. Despite conservative therapies, her knee pain worsened. She was barely able to crawl to the bathroom. Two weeks after the final injection, she presented to our hospital in a wheelchair.

### 2.3. Clinical Findings

**Figure 3:**
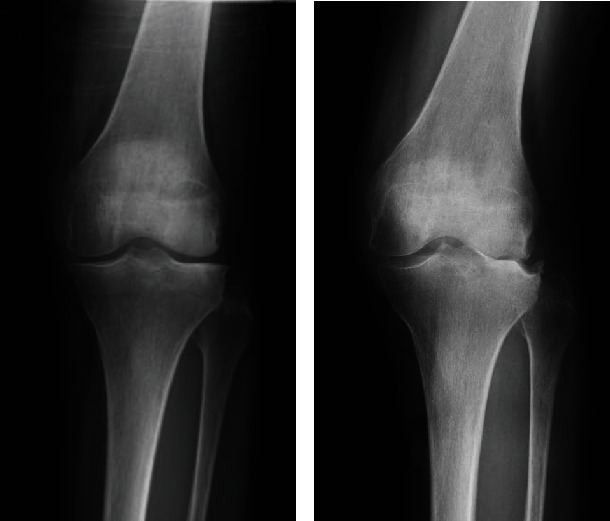
Preoperative radiographs of the left knee. (a) Radiograph at the first visit to our hospital shows Kellgren-Lawrence grade II and mild osteoarthritis of the lateral joint surface. (b) Radiograph at 1 month later shows Kellgren-Lawrence classification grade IV and severe valgus osteoarthritis.

The painful knee felt warm without swelling. A radiograph revealed moderate lateral osteoarthritis of the knee (Kellgren-Lawrence classification grade II) (Figure 3(a)). The range of motion preoperatively was 20 to 110 degrees. After repeated swelling and subsidence of the knee, the radiograph revealed progression to Kellgren-Lawrence grade IV in only 1 month (Figure 3(b)). Her white blood cell count and levels of C-reactive protein and procalcitonin were within normal limits. The erythrocyte sedimentation rate at the time of 60 minutes was 17 mm. Bacterial culture of the synovial fluid was negative. Candida and tuberculosis tests were also negative.

**Figure 4:**
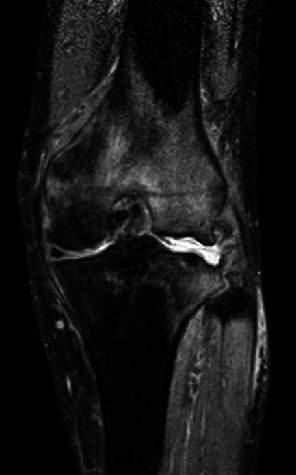
MRI of the left knee. Magnetic resonance image revealing partial depression of the joint surface including shredded ossicles and substantial amounts of synovial fluid. The findings were similar to those of osteomyelitis.

Magnetic resonance imaging (MRI) revealed partial depression of the joint surface including shredded ossicles and substantial amounts of synovial fluid. The T2-weighted coronal image revealed diffuse high-signal intensity, which suggested extensive spreading edema of the bone marrow and soft tissue (Figure 4). The change was considered to be caused by a SIF.

Bone mineral density measured at the femoral neck was 0.41 g/cm^2^. The *T*-score was very low (-3.5), suggesting severe osteoporosis.

### 2.4. Diagnostic Assessment

Based on the physical findings, laboratory data, and imaging findings on admission, rheumatoid arthritis and pyogenic arthritis were ruled out. Although there are few reports about Charcot spine and Charcot foot associated with PD [9–11], to the best of our knowledge, no publications have reported Charcot joint of the knee related to PD. It was therefore very difficult to arrive at the hypothesis that PD was the basal disease causing Charcot's joints. Yamamoto and Bullough concluded that SIF is the primary event leading to spontaneous osteonecrosis with histopathological study. Moreover, the histopathological findings of SIF are similar to those of spontaneous osteonecrosis [12]. However, the destruction of the joint surface was too rapid to enable the diagnosis of spontaneous osteonecrosis of the knee.

Finally, we diagnosed her with SIF of the knee on the basis of rapidly progressing destructive osteoarthritis within only a month. The MRI findings also supported the diagnosis of SIF. The SIF was also considered to be an initial symptom of steroid arthropathy because it occurred after intra-articular hydrocortisone injection. Steroid arthropathy was first described by Chandler et al. and is characterized by rapidly destructive osteoarthritis [13]. Hydrocortisone induces cartilage degeneration and bone atrophy. Pain relievers cause lack of perception feedback, due to which excess loads are often applied to knee joints. Steroid arthropathy of the knee is understood to occur as a result of pathological osteochondral fractures caused by increased mechanical stress [14, 15]. Steroid arthropathy needs to be identified with idiopathic osteonecrosis clinically and histopathologically. In this case, we did not perform the histopathological examination and could not certify the mechanism of SIF. However, rapid destructive steroid arthropathy might be conceivable to make the SIF.

### 2.5. Treatment Plan

Osteotomy or bone grafting would not have sufficiently reduced the pain and osteochondral remodeling because the joint surface was extensively destroyed and there was severe valgus alignment. Considering the patient's age, the level of activity, and the advanced stage of PD, total knee arthroplasty (TKA) was performed under general anesthesia.

### 2.6. Therapeutic Intervention

**Figure 5:**
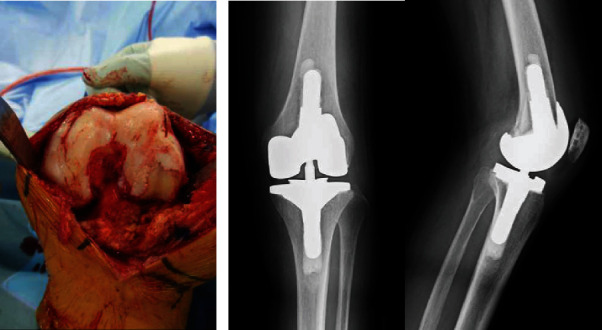
TKA. (a) Intraoperative photograph showing that the cartilage of the lateral joint surface of her left knee was scooped out and eburnated partially. (b) Postoperative radiographs of left TKA using a semiconstrained prosthesis.

TKA was performed using the medial parapatellar approach under general anesthesia. More bone than usual was resected because there was a severe extension contracture. Because the extension gap was tight on the lateral side, iliotibial band resection was added. There were severe bone atrophy and fragility in the medial epicondyle of the femur. The surface of the cartilage easily depressed under finger pressure. The cartilage of the lateral joint surface was scooped out and partially eburnated (Figure 5(a)). TKA was performed using a stemmed semiconstrained prosthesis (Attune Revision, DePuy Synthes, Warsaw, IN) because of concern for the failure of the medial collateral ligament caused by severe bone fragility in the medial epicondyle of the femur (Figures 5(b) and 5(c)). There were a satisfactory intraoperative implant gap, joint balance, and patella tracking. Prior to surgery, the poor mobility in her hip joint was thought to be affected by rigidity related to her PD. However, the poor mobility did not improve under general anesthesia. Her adductor longus tendon was resected because there was a hip adduction contracture. There was no particular restriction after treatment.

### 2.7. Follow-Up and Outcome

Her gait posture improved; however, postural deformities remained. There were no complications during hospitalization. There has been no early loosening of the prosthesis at the final follow-up: fifteen months after TKA.

**Figure 6:**
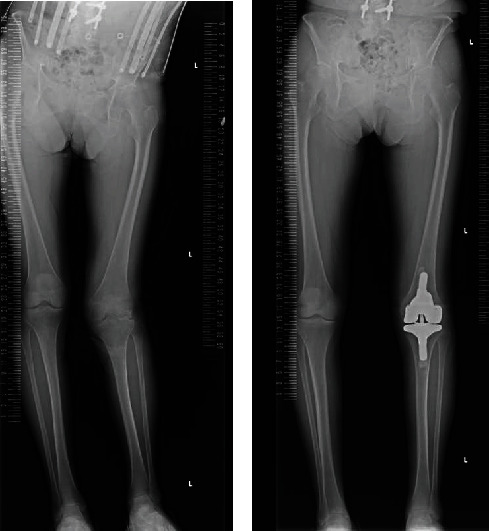
Total alignment of the lower extremity while standing. (a) Radiograph revealing preoperative total alignment of the lower extremity in a standing position. (b) Radiograph revealing postoperative total alignment of the lower extremity in a standing position.

She was able to walk with a cane and without knee pain. Her preoperative visual analogue score of 100 mm changed to 0 mm postoperatively. The preoperative range of motion was 25 to 110 degrees. This improved to 5 to 130 degrees postoperatively. A mild flexion contracture remained. The total alignment of the lower extremity improved dramatically after TKA and resection of the adductor longus tendon of the hip. The preoperative hip-knee-ankle angle (HKA angle) (-16 degrees while standing) was corrected to postoperative HKA angle (-2 degrees; positive values indicate the varus direction) (Figure 6).

## 3. Discussion

SIFs occur frequently at femoral heads or femoral medial epicondyles in osteoporotic older women [16, 17]. A SIF often develops with minor trauma. Some cases can heal with conservative non-weight-bearing. Early diagnosis with MRI should be done when the patient has severe pain that does not fit the radiographic images. In this case, we performed MRI after rapid destruction of the knee joint was detected. The findings of MRI suggested SIF of the lateral femoral epicondyle and lateral tibial joint surface. SIFs typically occur in osteoporotic thin middle-aged women. However, other predisposing factors are unclear.

Our patient had severe PD, a common neurodegenerative disease characterized by a kinematic tetralogy: akinesia/bradykinesia, rigidity, tremor, and loss of postural reflexes [18]. In recent years, postural deformities and freezing have been added to the symptom collection, bringing the total to six major symptoms of PD [19].

Pisa syndrome is a lateral flexion of the trunk that is frequently associated with long-standing PD [20, 21]. The postural deformity in PD is not based on the spinal deformity itself but on the loss of postural reflexes and the imbalance of muscle tonus. Our patient managed to remain standing and walk with a cane, leaning her body 10 degrees or more to her left side (Figures 1 and 2(c)). The leaning of the body exacerbated when walking but improved in the supine position. The postural deformity (Pisa syndrome) became noticeable when her PD symptoms had waned. The change of central sacral vertical line apical translation indicated the deterioration of Pisa syndrome after L1-L4 PLIF (Figure 2). In other words, the L1-L4 PLIF might have been worse Pisa syndrome. The more the trunk tilts to the lateral side, the center of the gravity axis, which is the mechanical axis, will shift and pass through more lateral points of the knee. Additionally, the weakness of the hip abductor muscle also led to lateral movement of the trunk away from the support limb, resulting in higher knee load [22]. Some studies indicate that trunk lean is associated with coronal knee moment [23–26]. The stress concentration from the spine to the lateral joint of the knee caused lateral knee osteoarthritis. Her knee pain appeared 1 month after L1-L4 PLIF as her Pisa syndrome worsened. Because there was no knee pain prior to the L1-L4 PLIF, Pisa syndrome was thought to have increased the load stress and bad effect on her knee joint.

The spine and hip joints are adjacent and therefore significantly affect each other. Offierski and MacNab first reported this in 1983 and termed it hip-spine syndrome [1]. Several studies have reported the relation between spinal alignment disorder and hip osteoarthritis [2–4]. Subsequently, it was discovered that apart from the adjacent hip joint, the knee joint equally affects spinal alignment [27–29]. In recent years, the syndrome is called knee-hip-spine syndrome [30].

Knee-spine syndrome is defined to occur when the preceding knee osteoarthritis with range of motion limitation leads to degenerative changes and three-dimensional kinematic imbalance of the trunk [5–7]. In case of unilateral osteoarthritis of the knee, as the flexion contracture worsens, the center of gravity shifts to the healthy side. The compensatory shifts of the center of gravity contribute to the onset of spinal disorders [7].

In our report, the patient had antecedent spinal postural disorder and the adverse effects of the same resulted in SIF and knee osteoarthritis. Considering this, this syndrome in this patient can be technically termed spine-knee syndrome.

Sato et al. reported that correction surgery for adult spinal disorder improves not only spinopelvic alignment but also the three-dimensional alignment of the lower extremities [31]. However, correction surgery in a patient with loss of postural reflexes and imbalance of muscle tonus can induce unexpected adverse events. A careful follow-up is essential for not only the spinal alignment but also the hip and/or knee joints.
